# Heat Stress-Induced Metabolic Remodeling in *Saccharomyces cerevisiae*

**DOI:** 10.3390/metabo9110266

**Published:** 2019-11-05

**Authors:** Daqiang Pan, Nils Wiedemann, Bernd Kammerer

**Affiliations:** 1 Centre for Integrative Signalling Analysis (CISA), University of Freiburg, 79104 Freiburg, Germany; daqiang.pan@mail.zbsa.uni-freiburg.de; 2 Institute of Pharmaceutical Science, University of Freiburg, 79104 Freiburg, Germany; 3 Institute of Biochemistry and Molecular Biology, ZBMZ, Faculty of Medicine, University of Freiburg, 79104 Freiburg, Germany; 4 CIBSS Centre for Integrative Biological Signalling Studies, University of Freiburg, 79104 Freiburg, Germany; 5 BIOSS Centre for Biological Signalling Studies, University of Freiburg, 79104 Freiburg, Germany; 6 Spemann Graduate School of Biology and Medicine (SGBM), University of Freiburg, 79104 Freiburg, Germany

**Keywords:** heat stress, yeast, metabolic remodeling, mitochondria, arginine metabolism, metabolomics

## Abstract

Yeast cells respond to heat stress by remodeling their gene expression, resulting in the changes of the corresponding proteins and metabolites. Compared to the intensively investigated transcriptome and proteome, the metabolic response to heat stress is not sufficiently characterized. Mitochondria have been recognized to play an essential role in heat stress tolerance. Given the compartmentalization of the cell, it is not clear if the heat stress-induced metabolic response occurs in mitochondria or in the cytosol. Therefore, a compartment-specific metabolite analysis was performed to analyze the heat stress-induced metabolic response in mitochondria and the cytoplasm. In this work, the isolated mitochondria and the cytoplasm of yeast cells grown at permissive temperature and cells adapting to heat stress were subjected to mass spectrometry-based metabolomics. Over a hundred metabolites could be identified, covering amino acid metabolism, energy metabolism, arginine metabolism, purine and pyrimidine metabolism, and others. Highly accumulated citrulline and reduced arginine suggested remodeled arginine metabolism. A stable isotope-labeled experiment was performed to analyze the heat stress-induced metabolic remodeling of the arginine metabolism, identifying activated *de novo* ornithine biosynthesis to support arginine and spermidine synthesis. The short-term increased spermidine and trehalose suggest their important roles as heat stress markers. These data provide metabolic clues of heat stress-induced metabolic remodeling, which helps in understanding the heat stress response.

## 1. Introduction

Heat stress is one of the most common environmental stress factors that yeast cells have to adapt to during their growth. Yeast cells respond to heat stress by remodeling their gene expression. They induce hundreds of genes related to cell stress, carbohydrate, and energy metabolism, and represses hundreds of genes related to translation and protein synthesis [1,2]. As a result of the altered transcription, 234 proteins were significantly changed after heat stress treatment by shifting the yeast growth temperature from 24 °C to 37 °C for 30 min [3]. Given the altered transcriptome and proteome upon heat stress, it is reasonable to expect an affected metabolome. Strassburg et al. [4] have investigated both transcriptomic and metabolomic responses of yeast adapting to temperature stress, identifying 38 altered metabolites. Based on a quantitative 1H NMR approach [5], 28 metabolites showed different concentrations between yeast grown at 30 °C and 37 °C, from which 25 belong to amino acid metabolism, nucleotide metabolism, respiration, and pyruvate metabolism. Since mitochondria play a central role in respiration and amino acid metabolism, mitochondria could contribute to the survival of yeast adapting to heat stress. Rikhvanov et al. [6] proposed that mitochondria are a central element of the heat stress response by showing reduced thermotolerance in oxidative growing yeast cells exposed to a mitochondrial inhibitor. Similar to cytosol, mitochondria have their own chaperone systems, conferring a compartment-specific thermotolerance [7]. Therefore, we hypothesize that heat stress-induced metabolic responses may be compartment-specific, as well.

Although metabolomics has been applied to measure the metabolite levels in cells or tissues [8,9,10], there has been a difficulty for a long time in analyzing the mitochondrial metabolites due to the subcellular compartmentalization and insufficient techniques. In the past few years, several methods have been developed to analyze the mitochondrial metabolome. The accurate concentrations of over hundred matrix metabolites could be measured by applying immunoprecipitation isolated mitochondria to metabolomics [11]. Furthermore, compartment-specific distribution and regulation of metabolites was observed in mammal [12] and yeast [13] cells by analyzing isolated mitochondria. These results provide new opportunities to discover subcellular metabolic disorders, resulting from diseases or environmental stresses.

Here, the isolated mitochondria and the cytoplasm from yeast cells adapting to heat stress were analyzed by mass spectrometry-based metabolomics, identifying over a hundred metabolites. By replacing arginine with the stable isotope-labeled form, a remodeled arginine metabolism was detected, implying its role for the heat stress response. These results display compartment-specific metabolic responses to heat stress in yeast cells, demonstrating the important role of mitochondria in cell stress tolerance.

## 2. Results and Discussion

### 2.1. Heat Stress-Induced Metabolic Responses Are Compartment-Specific

**Figure 1:**
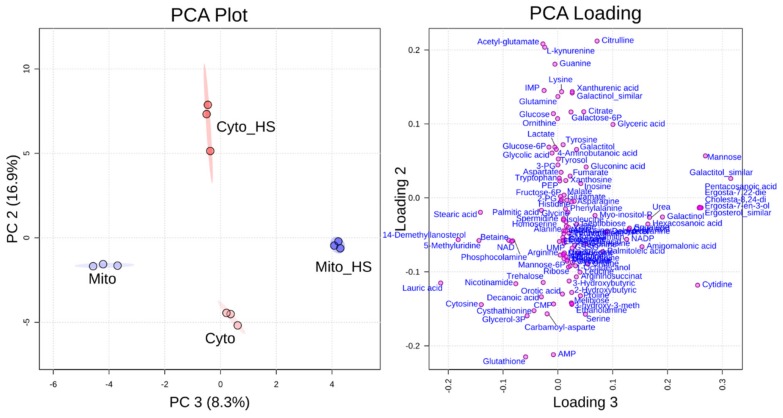
Principal component analysis (PCA) and loading plot. PCA (left) could separate metabolite levels of mitochondria (Mito) from mitochondria isolated after heat stress (Mito_HS) in component 3 and metabolite levels of the cytoplasm (Cyto) from cytoplasm isolated after heat stress (Cyto_HS) in component 2. PCA loading (right) shows how the single metabolites contributed to the separation of each group.

Yeast cells from the common lab strain BY4741 were grown on medium with galactose as a carbon source to induce a respiratory growth behavior which depends on the mitochondrial respiratory chain with an upregulated mitochondrial protein content [14,15]. To analyze the compartment-specific metabolite response to heat stress, cells were grown in liquid culture at 30 °C or additionally for 8 h at 37 °C. Subsequently, yeast cells were lysed by zymolyase treatment and pottering to separate mitochondria and the corresponding cytoplasm with buffers compatible for liquid chromatography-mass spectroscopy (LC-MS) metabolite analysis [11]. The mitochondrial fraction (Mito) and the corresponding cytoplasm (Cyto) from yeast cells grown at 30 °C were compared to the mitochondrial fraction (Mito+HS) and the cytoplasm (Cyto+HS), respectively, after heat stress (8 h at 37 °C). All the abbreviations of the samples will be used throughout this work. Samples were subjected to metabolic analysis in technical triplicate and the results have been reproduced by analyzing different biological preparations. Over a hundred metabolites could be identified by gas chromatography-mass spectrometry (GC-MS) and LC-MS in the mitochondrial and cytoplasmic fractions altogether, covering amino acids, energy metabolism, arginine metabolism, purine and pyrimidine metabolism, and lipid metabolism. By applying the data to principal component analysis (PCA), all four samples could be efficiently separated from each other. As shown in Figure 1, mitochondria isolated from cells grown at normal temperature could be separated from mitochondria isolated from heat-stressed cells through component 3, while the cytoplasm from cells under normal growth could be differentiated from heat-stressed cells through component 2. The PCA loading plot was generated to show how each single metabolite contributed to the discrimination in each component. As displayed on this plot, citrulline, acetyl-glutamate, glutathione and adenosine monophosphate (AMP) had a major contribution for the discrimination of component 2, while several lipids contributed to the discrimination of component 3. The detailed values of the loading plot can be found in Supplementary Table S1. The different alteration of metabolites in mitochondria and cytoplasm indicates a compartment-specific metabolic response to heat stress.

### 2.2. Lipid Metabolism

**Figure 2:**
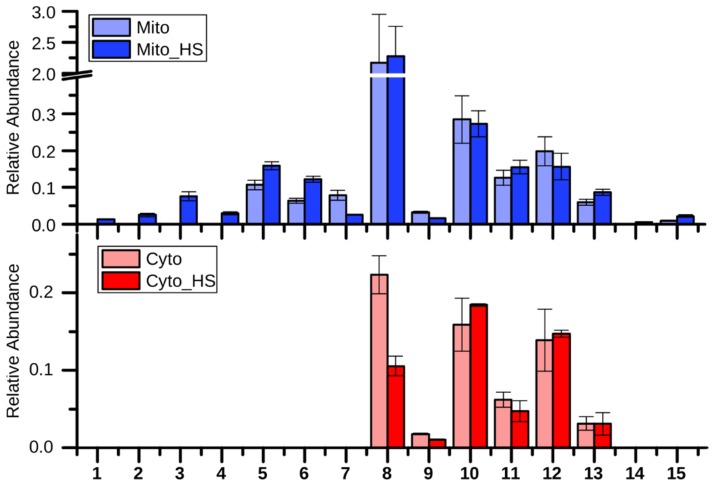
Relative abundance of the detected metabolites of lipid metabolism (mean +/- standard derivation of technical triplicate, applied also to other bar charts). **1**. Ergosta-7-en-3-ol; **2**. Cholesta-8,24-dien-3-ol, (3β,5α)-; **3**. Ergosta-7,22-dien-3-ol, (3β,22E)-; **4**. Ergosterol_similar; **5**. Lanosterol; **6** Squalene; **7**. 14-Demethyllanosterol; **8**. Ergosterol; **9**. Lauric acid (12:0); **10**. Palmitic acid (16:0); **11**. Palmitoleic acid (16:1); **12** Stearic acid (18:0); **13**. Oleic acid (18:1); **14**. Pentacosanoic acid (25:0); **15**. Hexacosanoic acid (26:0).

As observed in the loading plot (Figure 1), several lipids and fatty acids had a major contribution to the separation of stressed from non-stressed mitochondria (component 3). Interestingly, these lipid related metabolites, including several ergosterol derivatives (numbered **1**, **2**, **3**, **4** as shown in Figure 2) and pentacosanoic acid (25:0, **14**), could be detected only in mitochondria isolated from heat-stressed cells. While two ergosterol biosynthesis intermediates, lanosterol (**5**) and squalene (**6**), were increased upon heat stress, one further intermediate, 14-demethyllanosterol (**7**), was reduced. The final product, ergosterol, was not altered in the mitochondria but decreased in the cytoplasm. Ergosterol, as the major sterol in yeast membranes, has been shown to be related to stress tolerance [16]. Yeast with higher ergosterol levels presented better tolerances to heat and ethanol treatment through the increased membrane rigidity [17]. Therefore, it is reasonable to assume that the yeast cells remodeled the lipid composition to adapt to heat stress. Although the long chain fatty acids (**10**, **11**, **12**, **13**) were not significantly altered in both the mitochondria and the cytoplasm after heat stress, two very long chain fatty acids (VLCFAs), pentacosanoic acid (25:0, **14**) and hexacosanoic acid (26:0, **15**), were elevated in mitochondria after heat stress. VLCFAs are components of sphingolipids, which play an important role in signal transduction and protein transport [18,19,20] as well as in heat-induced cell cycle arrest [21]. Sphingolipid biosynthesis has been reported to increase upon heat stress [19], correlating with the elevated VLCFAs in our study.

### 2.3. Purine and Pyrimidine Metabolism

**Figure 3:**
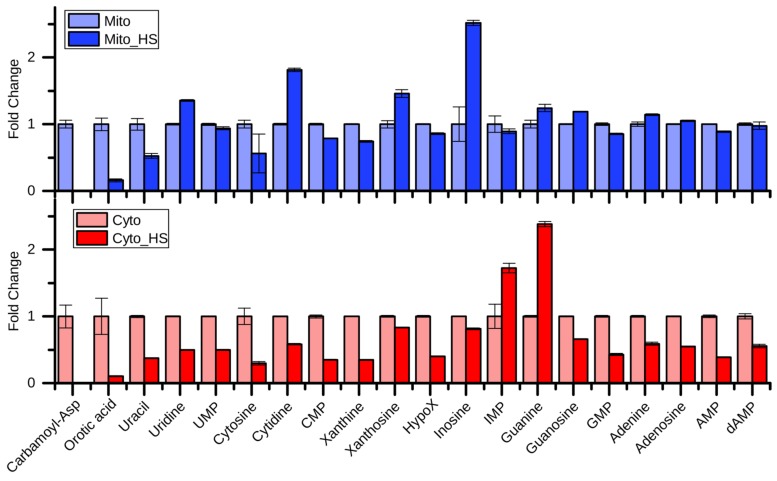
Fold change of purine and pyrimidine metabolism related metabolites after heat stress. Carbamoyl-Asp: carbamoyl-aspartate; HypoX: Hypoxanthine.

Although most of the purine- and pyrimidine-related metabolites were reduced in the cytoplasm after heat stress, their levels were maintained in mitochondria (Figure 3). While carbamoyl-aspartate, orotic acid, and uracil showed similar regulation patterns in mitochondria and the cytoplasm, uridine, cytidine, xanthosine, and inosine showed opposing regulation, suggesting a compartment-specific regulation of purine and pyrimidine metabolism intermediates upon heat stress. Surprisingly, carbamoyl-aspartate and orotic acid were highly depleted upon heat stress, indicating an interrupted *de novo* pyrimidine biosynthesis. Nevertheless, the product of this pathway UMP was maintained, especially in mitochondria. Given the upregulated cytidine and uridine, the UMP may be produced through a salvage pathway to maintain the mitochondrial DNA replication [22]. Interestingly, the most highly increased metabolites in the mitochondria after heat stress were inosine, cytidine, uridine, and xanthosine, which represent nodes of the salvage pathway, supporting the previous hypothesis. In contrast, guanine and IMP were highly increased in the cytoplasm after heat stress. IMP also represents a node in the purine nucleotide biosynthesis and salvage pathway.

### 2.4. Remodeling of Arginine Metabolism Upon Heat Stress

**Figure 4:**
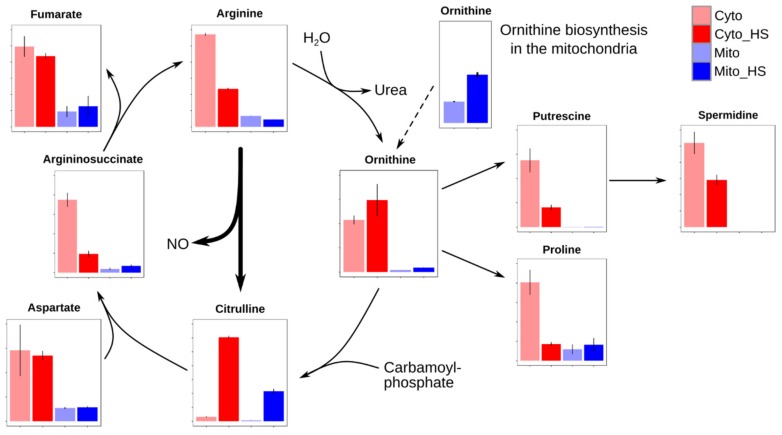
Relative abundance of arginine and related metabolites. The Y-axis indicates the relative abundance of each metabolite. Citrulline was highly accumulated after heat stress, while cytoplasmic argininosuccinate and arginine were reduced, indicating severely impaired arginine metabolism. The reason could be the direct conversion of arginine to citrulline, producing NO and leaving ornithine not dramatically accumulated.

As shown in Figure 4, citrulline was highly accumulated in the cytoplasm of heat stress-treated cells, explaining its dominant contribution to the discrimination of component 2 (Figure 1). Compared to the highly accumulated citrulline, its products, argininosuccinate and arginine, were four or two folds reduced under heat stress, indicating a severely impaired arginine metabolism. The reason for this could be either an interrupted catabolism or an increased influx of citrulline. Given that the reactant for argininosuccinate, aspartate, and the byproduct of arginine, fumarate, were both unaffected, it is questionable to expect an interrupted catabolism. Therefore, the decrease of arginine and the increase of citrulline can be the result of the activated nitric oxide (NO) production in response to heat stress. NO, as a cellular signaling molecule, has been reported to be related to stress tolerance by utilizing arginine to produce NO and the byproduct citrulline [23,24,25,26].

**Figure 5:**
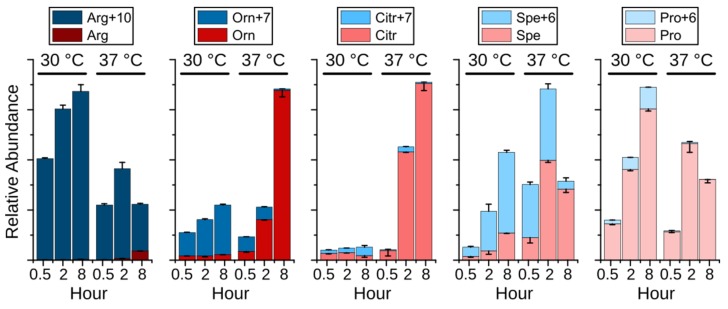
Relative abundance (mean +/- standard derivation, the error bars are displayed for the upper portion in plus and the lower portion in minus) of labeled arginine (Arg+10) and nonlabeled arginine (Arg) and the related metabolites labeled ornithine (Orn+7) and ornithine (Orn), labeled citrulline (Citr+7) and citrulline (Citr), labeled spermidine (Spe+6) and spermidine (Spe), labeled proline (Pro+6) and proline (pro). The cells were grown at either 30 °C or 37 °C for 0.5, 2, or 8 h.

To investigate the hypothesis above, an experiment was performed employing stable isotope labeled ^13^C_6_- and ^15^N_4_-labeled Arginine (Arg+10). Surprisingly, the isotopologues of arginine, ornithine, spermidine, and proline were dramatically changed upon heat stress. As displayed in Figure 5, Arg+10 was decreased at 37 °C, whereas nonlabeled arginine (Arg), originating from *de novo* synthesized ornithine, was detected after 2 h and increased after 8 h, indicating an increased biosynthesis and demand for arginine. Ornithine (Orn+6) was mainly produced from Arg+10 at 30 °C through eight hours, while its *de novo* synthesis (Orn) was activated at 37 °C. After 8 h at 37 °C, the *de novo* synthesis became the sole source of ornithine despite the presence of Arg+10, supporting the possibility of the conversion of arginine to citrulline during the NO production. Similar as in Figure 4, citrulline maintained at a low level at 30 °C. Interestingly, the elevated non-labeled citrulline (Citr) correlated with *de novo* produced ornithine, demonstrating the activated arginine biosynthesis upon heat stress. Although both proline and spermidine were synthesized from ornithine, they showed completely different metabolic fate upon heat stress. Compared to the immediately depleted Pro+6, the level of Spe+6 was significantly elevated after 0.5 h and 2 h, indicating an enhanced spermidine biosynthesis. Together with the reduced Arg+10 and Orn+7, the short-term increased spermidine (both Spe+6 and Spe) suggests that spermidine acts as a heat stress responder that could contribute to the survival of yeast under heat stress. Polyamines, including putrescine, spermidine, and spermine, have been shown to be essential for the growth of yeast cells, especially at 37 °C [27]. In addition, polyamines are involved in heat shock response in mammalian [28] and plant cells [29] by supporting expression of heat shock-related genes, suggesting a conserved function of polyamines in protecting cells from heat stress. However, the mechanism of spermidine as heat stress responder is still unclear. Overall, due to the conversion of arginine to citrulline, the *de novo* synthesis of ornithine was activated to support the arginine as well as the spermidine biosynthesis.

To evaluate the contribution of gene expression on the metabolic remodeling of the arginine metabolism, we analyzed published proteomic data dealing with yeast cells under heat stress. The observed protein amounts of arginine metabolism-related proteins were extracted from the supplemental table of Nagaraj et al. [3]. Interestingly, seven out of nine arginine biosynthesis proteins were upregulated, from which four had a fold change greater than 1.9 (Arg5,6 & Arg8: 2.3-fold, Arg3: 2.1-fold, Cpa1: 1.9-fold) [3]. This correlates with our results that arginine biosynthesis was stimulated under heat stress. In addition, arginine degradation enzymes, arginase (Car1) and ornithine transaminase (Car2), were increased by 1.5- and 3.7-fold, respectively [3]. Ornithine decarboxylase (Spe1), catalyzing the first step in spermidine biosynthesis, was highly upregulated (6.2-fold) upon heat stress [3], explaining the increased production of spermidine. However, two other required enzymes, S-adenosylmethionine decarboxylase (Spe2) and spermidine synthase (Spe3), were not significantly increased (1.3- and 1.0-fold, respectively) [3] indicating that their levels are not rate limiting. The fact that the proteomic data was generated using a different laboratory yeast strain and different growth conditions as the present study, suggests a general relevance for the remodeled arginine metabolism under heat stress identified in this study.

### 2.5. Trehalose

**Figure 6:**
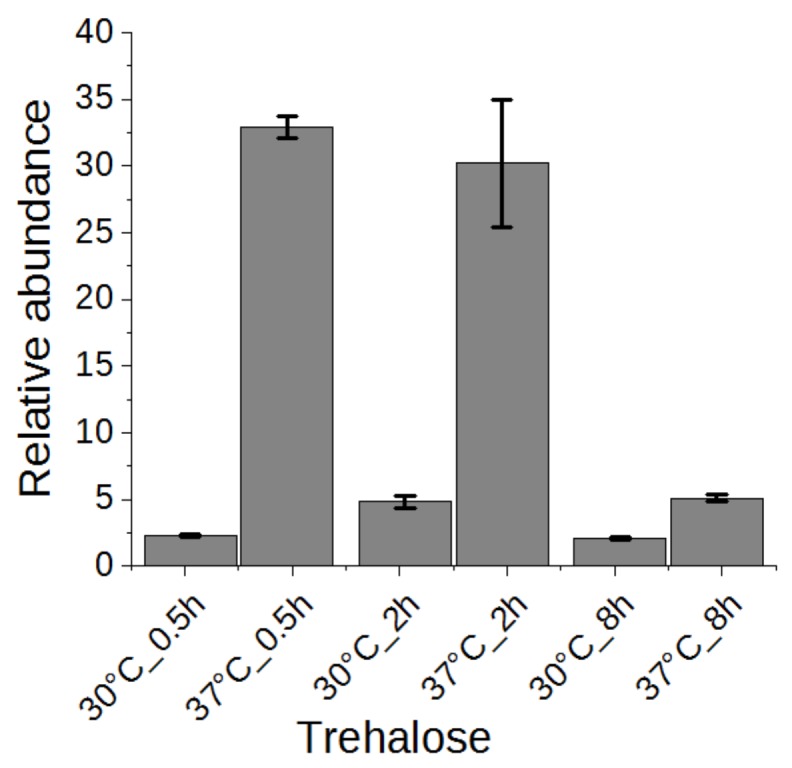
The change of trehalose in yeast cells under heat stress for 0.5, 2, or 8 h.

Trehalose has been suggested to be related to heat stress tolerance in yeast cells [1,2]. Thus, we examined the change of trehalose in a time series experiment. As shown in Figure 6, trehalose was highly accumulated in yeast cells upon heat stress after half an hour and maintained at a high level after two hours, conferring its role as heat stress response marker. After eight hours, the trehalose level was reverted significantly to almost the level of yeast grown without heat stress. It has been proposed that high concentration of trehalose can stabilize proteins and membranes, enhancing the cell’s thermotolerance [30,31]. In addition to genes encoding trehalose synthases (*TPS1*, *TPS2*, *TPS3*, *TSL1*) [2], the neutral trehalase-encoding gene *NTH1* was also activated [1], explaining the initial accumulation and subsequent reduction of trehalose. However, a constitutive expression of trehalose transporter gene *AGT1* did not rescue the high temperature-based growth defect associated with *tps1*Δ or *tps2*Δ [32]. Therefore, the precise role of increased trehalose synthesis upon heat stress remains to be demonstrated.

## 3. Conclusions

**Figure 7:**
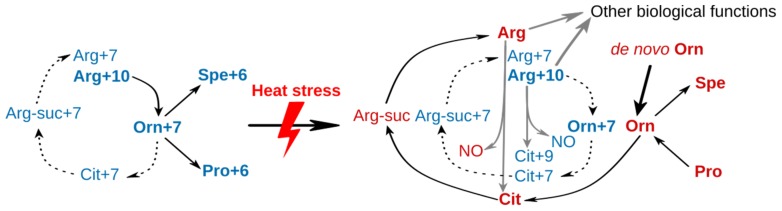
Remodeling of arginine metabolism pathway of yeast *Saccharomyces cerevisiae* upon heat stress. All labeled metabolites are shown in blue, while nonlabeled in red. The detectable metabolites are highlighted in bold text. Black arrow indicates the flux of a pathway, while grey arrow a putative pathway. Dash arrows indicate inactive pathways.

Although mitochondria have been recognized to have compartment-specific thermotolerance for over two decades [7], their metabolic role has not been studied in detail. In our study, a compartment-specific metabolomics approach was performed, revealing a large scale metabolic remodeling, including arginine, purine and pyrimidine, and lipid metabolism, in mitochondria and cytoplasm of yeast cells adapting to heat stress. The high accumulation of citrulline and reduction of arginine suggest an impaired arginine metabolism, which was proven by a stable isotope labeling experiment. Published proteomic data supports our model of activated arginine biosynthesis coupled with spermidine biosynthesis. As summarized in Figure 7, arginine was the major source of ornithine at 30 °C. No Arg+7 could be detected, indicating an inactive arginine synthesis through citrulline. This explains the low level of citrulline at 30 °C (Figure 4 and Figure 5). Upon sustained heat stress, the majority of ornithine was produced through *de novo* biosynthesis, instead of from arginine, to support spermidine biosynthesis, indicating a high demand for spermidine. Given the reduction of arginine and accumulation of citrulline, it is reasonable to assume that the NO biosynthesis was activated. Being a cellular signaling molecule, NO was shown to contribute to stress protection [33]. Moreover, the same alterations were observed in a rat model after receiving a heat treatment [34], conferring a conserved NO metabolism pathway in yeast and mammals. However, no Cit+9 could be detected at 37 °C. The reason for this could be that the NO production is coupled to the resynthesis of arginine. It has been reported that argininosuccinate lyase is required for NO production in both human and mice [35]. Although yeast *Saccharomyces cerevisiae* lacks the mammalian NO synthase ortholog, it is possible that similar mechanism exists in yeast. There has been evidence that yeast Tah18 has the NO synthase activity, producing NO in yeast cell upon stress [23,36]. However, the location of the NO production remains unclear due to the relocation of Tah18 upon heat or oxidative stress [36,37]. Our results indicate a new possibility to examine the NO synthase activity in yeast by investigating the interaction partners of the argininosuccinate lyase. Therefore, NO was probably produced merely through argininosuccinate (Arg-suc) synthesized Arg, instead of using Arg+10 from the medium. Although the cellular functions of spermidine and trehalose for thermotolerance are still not clear, their initial accumulation and subsequent reduction characterize them as heat stress markers. If and how spermidine and trehalose contribute to heat stress tolerance still need further investigation. Taken together, our data provide new insights into heat stress-induced metabolic remodeling, which helps in understanding heat stress response and tolerance in yeast *Saccharomyces cerevisiae*.

## 4. Materials and Methods

### 4.1. Yeast Culture

*Saccharomyces cerevisiae* BY4741 (Euroscarf, Oberursel, Germany) cells were cultured in liquid synthetic galactose medium (0.67% [*w*/*v*] yeast nitrogen base without amino acids (Becton, Dickinson and Company, Franklin Lakes, NJ, USA), 0.77 g/L SC amino acids (MP Biomedicals, Irvine, CA, USA), 2% [*w*/*v*] galactose (Sigma-Aldrich, Taufkirchen, Germany) under shaking at 130 rpm. For the stable isotope labeling experiment, arginine was replaced with the same concentration of L-arginine-^13^C_6_, ^15^N_4_ hydrochloride (Cambridge Isotope Laboratories Inc., Tewksbury, MA, USA).

### 4.2. Heat Stress Treatment

Yeast cultures were grown at 30 °C. For the final eight hours the temperature was maintained at 30 °C or shifted to 37 °C. Cells were harvested at the exponential phase.

For the stable isotope labeling experiment, cells were first grown to an OD_600_ of 2 at 30 °C before the same volume of medium, incubated at 30 °C or 44 °C, was added. The diluted cells were grown at either 30 °C as a control or at 37 °C as heat stress treatment. After 30 min and 2 h, cell culture, corresponding to 4 OD_600_ units, was directly added to 10 mL methanol, which was preincubated in dry ice for at least 2 h. For the 8 h time point, cells were cultured and collected similarly, except that cells were diluted to an OD_600_ of 0.1.

### 4.3. Mitochondria Isolation

Based on a previous protocol [13], mitochondria were isolated using a LC-MS compatible KPBS isolation buffer (180 mM KCl, 10 mM KH_2_PO_4_ (pH 7.2)) [11]. Briefly, the harvested cells were first incubated in dithiothreitol buffer before being treated with zymolyase (Seigaku). The cells were washed and pottered in ice-cold KPBS buffer. Mitochondria and the corresponding cytoplasm (post mitochondrial supernatant) were separated by differential centrifugation. After protein concentration determination using Bradford assay (Roth), mitochondria were aliquoted into 1 mg aliquots. The corresponding cytoplasmic fraction was calculated and aliquoted based on the total amount of mitochondria and the total volume of cytoplasmic fraction. All samples were stored at −80 °C.

### 4.4. Metabolite Extraction, Metabolomics Analysis, and Data Evaluation

Quenching and extraction of yeast cells was done as described by Hartl et al. [38]. Metabolite extraction of isolated mitochondria and cytoplasm, gas chromatography-mass spectrometry (GC-MS) analysis and metabolite annotation were performed as described before [13]. Two targeted metabolite analyses, for amino acids and pyrimidine metabolism intermediates, were applied to complement the GC-MS analysis using a multiple reaction monitor (MRM) mode on a liquid chromatography-triple quadrupole instrument (Agilent Technologies). For the amino acids, the method was adapted from Lagies et al. [39]. Briefly, the dried sample pellets were resuspended in 100 µL water before 5 µL of each was injected to a BEH-amide column (150 × 2.1 mm, Waters, Milford, MA, USA) with the column temperature set to 40 °C and a flow rate of 0.5 mL/min. Buffer A was 0.1% formic acid in water while buffer B was 0.1% formic acid in acetonitrile. The following gradient was used: 90% B to 75% B in 3.5 min, 75% B till 5 min, 75% B to 50% B from 5 to 11 min, 50% B till 13 min, 50% to 90% B from 13 to 14 min, and 90% B till 19 min. For the pyrimidine metabolism intermediates, 5 µL of the resuspended samples were injected to a HSST3 C18 column (150 × 2 mm, Waters) with the column temperature set to 30 °C. The following gradient was used: 0% B for 2 min, 0 to 5% B from 2 to 5 min, 5 to 99% B from 5 to 9 min, 99% from 9 to 16 min, 99 to 0% B from 16 to 17 min, and 0% B from 17 to 25 min. The data was processed and evaluated using the software MS Quantitative Analysis (Agilent Technologies, Waldbronn, Germany). All MRM transitions are attached in Supplementary Table S2.

## Supplementary Materials

The following are available online at https://www.mdpi.com/2218-1989/9/11/266/s1, Table S1: PCA loading values for Figure 1, Table S2: MRM transitions of purine and pyrimidine metabolism, and amino acid metabolism, Table S3: data processing GC/LC-MS, Table S4: data of isotope labeling experiment.
